# Adverse Events and Subsequent Management Associated With Eustachian Tube Balloon Dilation

**DOI:** 10.1002/oto2.70

**Published:** 2023-08-09

**Authors:** Sarah Jeoung, Li‐Xing Man, Alexander K. Mandych, Isaac L. Schmale

**Affiliations:** ^1^ University of Rochester School of Medicine and Dentistry University of Rochester Rochester New York USA; ^2^ Department of Otolaryngology–Head and Neck Surgery University of Rochester Medical Center Rochester New York USA

**Keywords:** balloon dilation, Eustachian tube dysfunction, Manufacturer and User Facility Device Experience, subcutaneous emphysema

## Abstract

**Objective:**

Eustachian tube balloon dilation is a minimally invasive technique used to improve persistent Eustachian tube dysfunction. Currently, the US Food and Drug Administration (FDA) has approved the use of balloon dilation devices produced by three manufacturers, but little is known about associated adverse events and subsequent management.

**Study Design:**

Case series.

**Setting:**

FDA Manufacturer and User Facility Device Experience (MAUDE) database.

**Methods:**

Reports submitted to the FDA using the MAUDE database searched from January 2000 to July 2022 were analyzed for adverse events and management.

**Results:**

A total of 13 adverse events were found in the database. Subcutaneous emphysema (n = 8) was the most common event. Other less frequent events included patulous Eustachian tube (n = 2), vascular dissection (n = 1), nasopharyngeal mucocele (n = 1), and tinnitus (n = 1). A majority of patients who experienced subcutaneous emphysema received antibiotics (n = 5) and were admitted to the hospital (n = 4). The patient with a carotid dissection 7 days postprocedure presented with a stroke and fully recovered after stent placement. There was limited preprocedure information in the MAUDE database. There were 2 patients who did not fully recover after a complication. Three patients underwent corrective surgical interventions. No one company had more associated adverse events reported.

**Conclusion:**

Subcutaneous emphysema is the most common adverse event after Eustachian tube dilation. Further studies exploring potential balloon dilation adverse events to allow for better patient counseling are warranted.

It is estimated that Eustachian tube dysfunction (ETD) affects 4.6% of the adult population and 6.1% of the child population in the United States.^1^ ETD occurs when the Eustachian tube is unable to equalize pressure between the nasopharynx and middle ear, transport secretions and other material from the middle ear via mucociliary transport, and protect the middle ear from loud noises and potentially harmful substances by closing. The diagnosis of ETD is mainly based on subjective symptoms. According to the American Academy of Otolaryngology–Head and Neck Surgery 2019 clinical consensus statement on the use of balloon dilation, a combination of subjective and nonspecific patient complaints, a physical exam including the tympanic membrane, and office‐based nasal endoscopy is needed for an ETD diagnosis.^2^ This can help identify underlying extrinsic etiologies such as allergic rhinitis, rhinosinusitis, and laryngopharyngeal reflux which have targeted treatments that could lead to symptomatic relief of ETD.

Patients can experience a wide range of symptoms that include otitis media with effusion, tympanic membrane retraction and perforation, tinnitus, aural fullness, otalgia, autophony, and imbalance. Etiologies include baro‐challenge‐induced ETD, patulous Eustachian tube, and obstructive ETD.^2^ Repeated equalization maneuvers in both increased and decreased surrounding pressures can cause localized stress on the mucosal surface of the Eustachian tube and lead to inflammation and edema that can affect the structure's ability to open and clear the middle ear. Patients with patulous Eustachian tubes are unable to close their Eustachian tubes leading to constant communication between the middle ear and nasopharynx. Possible sources for obstruction include mucosal inflammation and edema (due to allergies, rhinitis, upper respiratory infection), adenoid hypertrophy, and malignancy.

To date, there is no accepted standard medical therapy for ETD described in the literature. Norman et al conducted a systematic review that found no benefit from ETD therapies, including management with observation, antibiotics, or nasal steroids.^3^ Another systematic review found minimal improvement in symptoms with intranasal corticosteroids and no evidence that one medical management option was more efficacious than others.^4^

In addition to the potential discomfort of untreated ETD, the lack of primary interventions and optimal treatment algorithm often creates unnecessary financial impacts for patients. It was estimated in 2019 that the overall annual cost for prescriptions for adult patients diagnosed with ETD totaled $8.5 million; over half was spent on antibiotics and intranasal steroids and the other half on analgesics including opioids.^5^ Invasive otologic and sinonasal procedures used for therapeutic relief including Eustachian tube inflation without cauterization and myringotomy with tympanostomy also add potentially unnecessary costs for patients. The estimated annual cost of otologic and sinonasal procedures for adult ETD patients amounted to $10.5 million and $35.3 million, respectively.^6^

Balloon dilation of the Eustachian tubes was first attempted around 2010. The marketing of a device that uses an endoscopically directed balloon to treat persistent obstructive ETD was approved by the US Food and Drug Administration (FDA) in 2016. Using a minimally invasive transnasal endoscopic method, a balloon catheter is guided into the Eustachian tube and transiently inflated. With a short postoperative recovery period and subjective long‐term clinical improvement for approximately two‐thirds of patients, this procedure has become an increasingly popular option for treating chronic ETD.^7^

There is little information about the adverse events associated with the procedure in the literature. Meyer et al conducted a randomized controlled trial comparing balloon dilation to continued medical therapy with 60 subjects. The authors of this study found statistically significant symptomatic improvement through 1‐year postdilation but did not identify adverse events.^8^ Prior documented adverse events include subcutaneous emphysema and hypoglossal paresis.^9^, ^10^ The literature shows an overwhelming benefit associated with balloon dilation compared to adverse events. However, it is vital to continue exploring all possible adverse events in order to improve clinical guidelines and understand patient safety issues. There remains a need for further studies to explore this question to better guide treatment for patients experiencing chronic ETD.

Currently, the FDA has approved of devices produced by 3 manufacturers: Acclarent Inc, Stryker Corp, and Medtronic plc. The FDA monitors device performance and possible safety issues through medical device reports (MDRs) submitted by voluntary and mandatory reporters. The voluntary reporters consist of health care professionals and patients while the mandatory reporters consist of manufacturers, importers, and facilities using the device. The Manufacturer and User Facility Device Experience (MAUDE) database houses the several hundred thousand MDRs submitted each year to help with risk‐benefit evaluations.

The purpose of this study is to review MDRs to provide insight into the observed adverse events associated with Eustachian tube balloon dilation and management.

## Methods

MDRs associated with balloon dilation devices produced by the 3 approved manufacturers submitted to the FDA were identified using the MAUDE database from January 1, 2000 to July 28, 2022. A search was conducted initially using the product codes “PNZ” and “LRC.” To ensure all relevant reports were included in the analysis, the term “Eustachian tube dilation” was used. A total of 15 reports were identified. Variables collected included event date, manufacturer, device, adverse events, hospitalizations, and interventions performed. The University of Rochester Research Subjects Review Board deemed this study as research not involving human subjects as defined by the Department of Health and Human Services and FDA regulations and exempted this study from further review and approval.

## Results

From the database, 13 adverse events and 2 device malfunctions were identified and further analyzed (Table 1). No mortalities were reported. A small subset of the patients did not fully recover from the sequelae of Eustachian tube balloon dilation (n = 2). The most common adverse event reported was subcutaneous emphysema (n = 8) involving the face, neck, and/or chest followed by a patulous Eustachian tube (n = 2). Vascular dissection (n = 1), nasopharyngeal mucocele (n = 1), and tinnitus (n = 1) were also reported. Most patients who experienced subcutaneous emphysema received antibiotics (n = 5) and hospital admission (n = 4). A small portion of patients improved with no intervention (n = 3). One patient had a chest tube placed in the intensive care unit after being diagnosed with subcutaneous emphysema of the right face, neck, and chest. The patient with vascular dissection presented to the Emergency Department with a stroke was diagnosed with a carotid dissection and had a stent placed. The patient made a full recovery. There is no information about preprocedural screening and measures written in the MDR. Out of the 2 patients who did not fully recover, one reported hearing loss and tinnitus 6 months postprocedure and the other remained distressed about their patulous Eustachian tube diagnosis and associated ongoing symptoms. There were no adverse events reported with devices produced by Medtronic, but these devices were relatively new to the market at the time of our search. Out of the other 2 manufacturers, no one company was found to have more reported adverse events.

**Table 1:** Adverse Events by Category

Patient	13
Subcutaneous emphysema	8
Patulous Eustachian tube	2
Vascular dissection	1
Tinnitus/hearing loss	1
Nasopharyngeal mucocele	1
Device	2
Unsealed	1
Material bent/ruptured	1

## Discussion

Balloon dilation for persistent ETD has become a mainstay of treatment and is considered to be effective with limited adverse events. To our knowledge, this is the first study to report device‐related adverse events of Eustachian tube balloon dilation utilizing a national database (MAUDE). A total of 13 patient‐related events were found with subcutaneous emphysema being the most reported. Among the 13 reported complications, 11 patients fully recovered with no sequelae while 2 had ongoing issues with patulous Eustachian tube symptoms.

There are multiple studies showing the long‐term benefit of balloon dilation for ETD through multiple measures. In the randomized controlled trial conducted by Meyer et al, around 66% of patients who underwent balloon dilation and had retracted tympanic membrane positioning at baseline had an improvement in position at 6 weeks postprocedure.^8^ The 7‐item Eustachian Tube Dysfunction Questionnaire (ETDQ‐7) is a validated measure to evaluate the severity of symptoms where a score less than 2 means no/mild symptoms, a score between 3 and 5 indicates moderate symptoms, and a score from 6 to 7 indicates severe symptoms. The authors found a significant drop in score after balloon dilation (average 4.6 to 2.1, *p* < .0001) at 6 weeks postdilation that was maintained at the 12‐month follow‐up.^8^ In another systematic review with 1155 patients, all types of evaluation including ETDQ‐7 scores, the Valsalva maneuver/Toynbee test, tympanometry, and audiometry showed both short‐term and long‐term improvement during an average of 6.9 months of follow‐up.^7^ However, literature regarding potential adverse events secondary to Eustachian tube dilation remains scarce.

One early study by Catalano et al reviewed 100 balloon dilations in 70 patients a few years after balloon dilation was first approved by the FDA.^9^ They performed the dilations and followed the patients from January 2009 to 2011. Ear fullness and pressure improved in 71% of patients who were followed for an average of 26.3 weeks, and 87% of the 8 patients who were followed for greater than 34 weeks reported symptom relief. One patient was found to have subcutaneous emphysema postoperative day 2, limited to the ipsilateral parotid region, that resolved spontaneously within 48 hours. The authors stated that the procedure is unlikely to cause patulous Eustachian tubes.

To date, no studies have explored patulous Eustachian tubes, nasopharyngeal mucoceles, or tinnitus as possible complications from balloon dilation. However, the results from the MAUDE database highlight these as rare complications (4 total cases reported to date). This highlights the importance of reporting on rare complications and intermittent checks on approved devices or procedures to ensure that patients are being appropriately counseled. There remains a need for further larger adverse event studies to better evaluate potential balloon dilation treatment complications and ensure patient safety.

The petrous portion of the carotid artery runs posterior to the bony portion of the Eustachian tube. Due to the anatomical proximity, previously a high‐resolution computed tomography (CT) scan was recommended preoperatively due to concerns for carotid injury with balloon dilation of the Eustachian tube. Abdel‐Aziz et al explored the value of preoperative high‐resolution CT to predict procedural difficulties and the possible complication of carotid injury. They reviewed 284 patients with preoperative CT scans of the temporal bone from January 2009 to December 2012 and found that 18 patients (6.3%) had carotid canal dehiscence. Three patients could not undergo the procedure due to difficulty advancing the catheter with one found to have bilateral carotid canal dehiscence. The study found 3 adverse events (2 instances of soft tissue emphysema and 1 unilateral hypoglossal paresis) that self‐resolved with associated unremarkable CT scans and no incidences of carotid dissection.^10^ They recommend against preoperative CT imaging due to rare complications due to carotid abnormalities.

Ockermann et al did a preliminary study on cadavers using high‐resolution CT scans to ensure no damage to nearby structures such as the carotid canal occurred during Eustachian tube dilation. They found no evidence of internal carotid artery injury but some mucosal lacerations near the opening of the Eustachian tube in the nasopharynx.^11^ Our study found 1 reported case of a stroke secondary to carotid injury without any reported sequelae. Given that carotid artery injury has affected patients undergoing balloon dilation, one must weigh the risks of radiation exposure and costs of CT imaging for many versus the potentially devastating complication for a few when counseling patients regarding Eustachian tube dilation and preprocedural investigations.

There are limitations to our review. First, the MAUDE database is made up of self‐reported claims not confirmed by the FDA and may contain incomplete, inaccurate, or biased data. No official reports such as medical records are included in the MAUDE database making it difficult to further explore the adverse events. Additionally, balloon dilation cannot be directly linked to certain complications due to a lack of standard reporting methods. Because this is a self‐reported database, we do not have information on the total number of patients who underwent the surgery during the study period. Further studies are necessary to ascertain incidence information for balloon dilation complications. There is no current data or recommendations on how to minimize the occurrences of possible complications of balloon dilation. Further study with more complications over time would be necessary to produce actionable advice to improve the safety of this procedure.

In this review, adverse events of balloon dilation of the Eustachian tube which had previously been sparsely or never reported were explored. Further knowledge about potential complications encountered during real‐world use is valuable to providers who perform the procedure to better counsel patients about the risk and benefits of available treatment options. Additionally, continual assessment of new procedures and devices is important to ensure safety concerns are not raised when used outside the setting of clinical trials.

## Conclusion

Overall, balloon dilation to address chronic ETD is a safe and effective treatment option for patients. Relatively few complications were reported compared to the number of procedures performed nationally. The most reported adverse event following Eustachian tube balloon dilation for ETD was subcutaneous emphysema. Most complications resolved without sequelae but a risk of persistent dysfunction after complications was possible. Given that carotid injury after balloon dilation for ETD has occurred, the costs and low risks associated with radiation exposure that comes with CT scans must be weighed against the potential benefit of carotid artery injury avoidance. This question along with the lack of diagnostic testing and standardized treatment approach should be further explored with additional studies to minimize harm when determining if balloon dilation is appropriate for patients.

## Authors Contributions

**Sarah Jeoung**, design, conduct, analysis, and presentation of research; **Li‐Xing Man**, design, review manuscript; **Alexander K. Mandych**, review manuscript; **Isaac L. Schmale**, review manuscript.

## Disclosures

### Competing interests

None.

### Funding source

None.
